# A Comparative Review of Donkey Genetic Resources, Production Traits, and Industrial Utilization: Perspectives from China and Globally

**DOI:** 10.3390/ani15233372

**Published:** 2025-11-21

**Authors:** Qifei Zhu, Muhammad Zahoor Khan, Yongdong Peng, Changfa Wang

**Affiliations:** College of Agriculture and Biology, Liaocheng University, Liaocheng 252000, Chinazahoorkhan@lcu.edu.cn (M.Z.K.)

**Keywords:** donkey genetic resources, production performance, donkey milk, welfare, sustainable development, conservation

## Abstract

The donkey (*Equus asinus*), despite its long domestication history, has often been overlooked and is currently facing the dual challenge of genetic erosion and emerging economic opportunities. Global research indicates that although Chinese indigenous breeds maintain a moderate level of genetic diversity, the rapid transition from draft purposes to large-scale ejiao production has driven them into severe endangerment, leading to a dramatic decline in donkey population. In contrast, donkey populations in Europe and the Mediterranean, though numerically smaller, have achieved greater stability through conservation programs and integration into specialized sectors such as dairy production and tourism. Genomic studies further reveal region-specific selection for traits associated with body size, adaptability, and productivity, underscoring the profound influence of ecological and management contexts. This review systematically compares Chinese and international donkey breeds in terms of genetic resources, genetic characteristics, phenotypic and reproductive traits, adaptive capacity, and industrial utilization models, with the aim of providing a foundation for global strategies in genetic conservation and sustainable development.

## Simple Summary

This comprehensive review examines global donkey genetic resources, production traits, and utilization models across China, Europe, Africa, and Mediterranean regions. Through systematic comparison of genomic diversity, morphological characteristics, and production systems, the review reveals distinct regional trajectories: European populations achieve stability through specialized dairy operations and conservation programs, African populations maintain traditional draft roles with high genetic diversity, while China transitions toward diversified commercial applications. Key findings include genomic selection signatures for body size and environmental adaptation, comparative analyses of milk composition and meat quality across breeds, disease resistance profiles, and industrial development patterns. The synthesis provides evidence-based strategies for integrating genetic conservation with sustainable utilization pathways globally.

## 1. Introduction

The donkey (*Equus asinus*), one of the earliest domesticated livestock species, has been integral to human societies for over 5000 years, providing essential contributions in labor, milk and meat production, hide processing, and cultural heritage across diverse civilizations [1,2]. While the traditional role of donkeys in draft work has diminished due to agricultural mechanization and socio-economic transitions, their economic and scientific value in milk production, meat and skin industries, companion animal services, and biopharmaceutical applications has garnered increasing recognition in the 21st century [3,4,5,6]. According to the Food and Agriculture Organization [7], the global donkey population is estimated at approximately 50 million head, with the majority concentrated in Asia and Africa, particularly within developing regions. Conversely, Europe and North America maintain comparatively smaller populations, prioritizing local breed conservation, specialized farming systems, and diversified functional applications [7,8,9].

In China, donkeys have historically occupied a central position in agricultural production and transportation infrastructure, resulting in the development of 24 indigenous breeds, including the Dezhou, Guanzhong, and Guizhou breeds [10]. However, rapid industrialization and evolving production systems have precipitated a substantial decline in donkey populations, accompanied by reduced genetic diversity and an elevated extinction risk among small local breeds [11]. In contrast, European and Mediterranean countries, despite maintaining smaller population sizes, have established comprehensive conservation frameworks through formal breed registries, systematic genetic monitoring programs, and the promotion of diversified utilization strategies—including donkey milk for human consumption and agritourism initiatives—thereby achieving a sustainable balance between conservation objectives and economic viability [12,13,14].

Genomic and archeological investigations have yielded valuable insights into donkey domestication history and evolutionary trajectories [15], while contemporary research has examined phenotypic variation in growth performance, reproductive traits, and adaptive capacity across breeds from different geographic regions [16,17,18]. Nevertheless, existing studies remain fragmented, often constrained to specific breeds or phenotypic traits, and lack a comprehensive international comparative framework. Given the ongoing transformation of the global livestock sector, there is an urgent need to develop integrated strategies that reconcile genetic diversity conservation with sustainable utilization of donkey genetic resources [19].

Accordingly, the present review aims to systematically compare domestic and international donkey breeds with respect to genetic resources, phenotypic and reproductive characteristics, and patterns of industrial utilization. By synthesizing current knowledge and elucidating differences in development trajectories and management models, we seek to provide a scientific foundation for future strategies in genetic conservation, performance enhancement, and multifunctional utilization, thereby contributing to the long-term sustainability of the global donkey industry.

## 2. Materials and Methods

A literature-based approach was employed to summarize global genetic and phenotypic diversity in domestic donkeys. Relevant studies published between 2009 and 2025 were retrieved from Google Scholar, Web of Science, and Elsevier using keywords related to donkey breeds, phenotypic traits, genetic diversity, and adaptive evolution. Inclusion criteria focused on studies providing primary data or comprehensive analyses of genetic backgrounds, phenotypic characteristics, adaptive mechanisms, or resource value, with final references selected through multi-stage screening for relevance and completeness.

## 3. Comparison of Germplasm Resources and Genetic Characteristics

**Figure 1:**
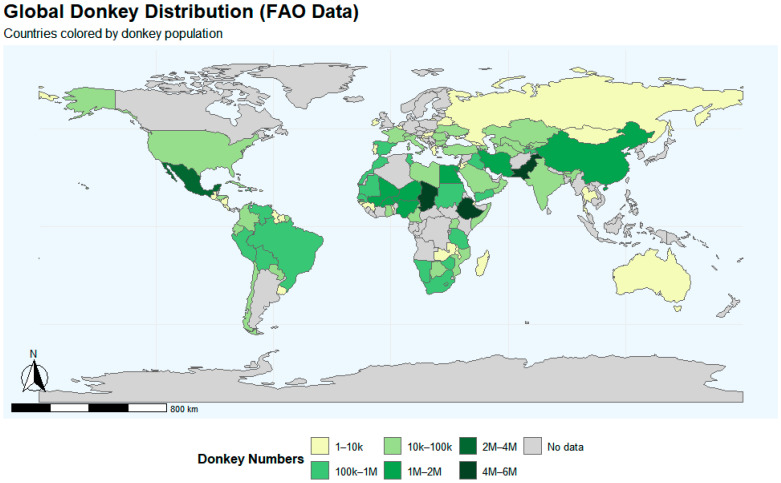
Global donkey population and distribution map. Data from FAO Animal Production and Health Statistics (accessed 12 August 2025); visualized in R.

Globally, donkey germplasm resources show marked variation in population size, distribution, and genetic structure [8,15,20]. Based on FAO Animal Production and Health Statistics, Figure 1 illustrates the uneven global distribution of donkeys, with high concentrations in Africa and Asia and smaller populations elsewhere. These patterns reflect long-term influences of ecological conditions and economic use, underscoring the challenges for conservation and sustainable utilization. This section offers a brief overview of global donkey germplasm resources, focusing on three key aspects: (i) a comparison of morphological traits between Chinese and international breeds, (ii) a review of genetic diversity and structure, highlighting differences in resilience and inbreeding risks, and (iii) an evaluation of conservation efforts, contrasting strategies and challenges globally. This structure places Chinese donkey breeds within the broader global context, emphasizing ecological, evolutionary, and industrial influences.

### 3.1. Comparative Morphological Characteristics of Chinese and International Donkey Breeds

Chinese donkey breeds display a continuum of body sizes and conformations, ranging from small-bodied plateau ecotypes to large-bodied meat-and-draft dual-purpose breeds (See Table 1). Large-framed breeds such as the Dezhou donkey have expanded under market-driven selection for the ejiao industry, whereas small-framed breeds, such as the Huaibei Gray donkey, have experienced demographic marginalization due to limited commercial value [21,22,23]. Internationally, European large-bodied breeds (e.g., Martina Franca, Andalusian) also exhibit enhanced stature but suffer from restricted population sizes, while some insular breeds (e.g., Graciosa donkey) display diminutive stature, reflecting intensive artificial selection and geographic isolation (Table 2) [7,24,25,26,27,28,29,30,31,32]. Thus, morphological diversification in China primarily reflects market-oriented selection, whereas in Europe and Mediterranean regions, it is strongly influenced by geographic isolation and historical breeding practices.

### 3.2. Comparison of Genetic Structure and Diversity Between Chinese and International Donkey Populations

**Figure 2:**
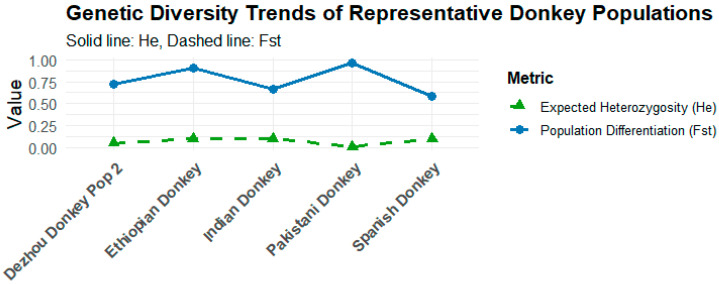
Trend of genetic diversity of representative donkey populations.

At the molecular level, Chinese donkey breeds maintain moderate to high within-population genetic diversity (H_e_ = 0.6315–0.6999), with low inter-population differentiation (F_ST ≈ 0.06), indicating that genetic variation is largely contained within populations [44] (Figure 2). Small local breeds, however, exhibit increased inbreeding (elevated F_IS), signaling potential genetic erosion [44,45]. In comparison, sub-Saharan African working populations retain higher genetic diversity and lower differentiation due to large population sizes and extensive gene flow [36,39], whereas European and insular populations are more prone to genetic drift and inbreeding, resulting from historical bottlenecks and geographic isolation [46]. Overall, Chinese populations occupy an intermediate genetic state, retaining diversity at the national level while experiencing localized contraction in smaller breeds, reflecting a dual pattern between African high-diversity equilibrium and European diversity-depleted populations [11,36,39,44,45,46,47].

### 3.3. Conservation Status of Chinese and International Donkey Breeds

Conservation status shows a marked contrast between China and other regions. In China, over half of the 24 indigenous breeds are currently classified under varying degrees of endangerment, particularly small local breeds such as the Huaibei Gray donkey [22,23]. While industrially favored large breeds benefit from structured breeding programs and germplasm repositories, many smaller breeds face rapid population decline and elevated inbreeding, necessitating integrated conservation strategies, including in situ protection, ex situ germplasm preservation, and molecular monitoring [11,44,45,47,48,49,50].

Internationally, African and South Asian donkeys maintain large, free-ranging populations with substantial genetic diversity, but lack formal monitoring and breed registration, leaving them vulnerable to genetic erosion despite census size [36,39]. European and Mediterranean breeds, in contrast, are well-documented and managed, but their small, isolated populations are prone to inbreeding and genetic drift, requiring proactive conservation measures such as controlled mating and ex situ preservation [7,26,30,46,51]. Comparatively, China faces a dual challenge: supporting industrial breeding of large, commercially valuable breeds while safeguarding small endangered local breeds, necessitating systematic, science-based management frameworks [11,22,23,47,48,49,50].

## 4. Comparative Analysis of Production Performance and Environmental Adaptability

The overall value of donkey genetic resources is determined by the interplay between production traits—including draft capacity, meat and milk yields, and reproductive efficiency—and environmental adaptability, such as tolerance to climatic stressors [52]. These trait complexes vary markedly among global donkey populations, reflecting adaptation to diverse agroecological conditions and influencing both economic performance and long-term sustainability [53,54,55]. In the context of genetic resource management, optimizing production traits enhances economic returns, while maintaining adaptive resilience is essential to ensure breed persistence and support targeted improvement programs.

### 4.1. Growth and Reproductive Traits

Donkey growth and reproductive performance vary widely across regions, reflecting interactions between genetics, environment, and management [56]. Large-framed European and American breeds typically achieve greater mature body size and slower growth under intensive systems, supporting high-value meat or breeding programs, but they require higher nutritional inputs and extended generation intervals [33,57]. In contrast, medium- and small-bodied indigenous breeds in Asia and Africa exhibit faster relative growth, earlier sexual maturity, and higher reproductive efficiency under extensive or semi-intensive systems, highlighting their adaptability to low-input conditions [10,58,59,60].

Reproductive dynamics further illustrate these trade-offs. Estrous cyclicity and gestation length are broadly conserved, yet conception rates and postpartum fertility are sensitive to management intensity [61,62]. Early postpartum breeding commonly results in lower conception across all regions, whereas estrus monitoring and ovulation synchronization improve reproductive outcomes [63,64]. These contrasts emphasize a recurring pattern: production-focused breeds may achieve higher outputs but are more management-dependent, whereas locally adapted breeds prioritize resilience and reproductive reliability under variable conditions.

### 4.2. Draft Capacity and Environmental Adaptation

Working performance demonstrates pronounced regional specialization. In South and Central Asia, donkeys carry exceptionally high relative loads (up to 77% of body weight) in short-distance transport, reflecting adaptations for muscular endurance under intensive labor [65,66,67,68]. Sub-Saharan African donkeys are optimized for long-distance water and goods transport, demonstrating heat tolerance and sustained stamina, while North African and Middle Eastern highland breeds emphasize stability and agility on steep, uneven terrain [54,68,69,70,71]. European breeds, less adapted to environmental extremes, are increasingly used in precision agriculture with moderate load demands [72]. Chinese donkeys often exhibit a hybrid pattern, retaining traditional draft abilities while contributing to meat, milk, and hide production [10,17,73].

This spectrum illustrates how functional specialization aligns with ecological and management pressures. High-load endurance is favored where labor demands are extreme, whereas stability, precision, or combined production traits dominate in other systems [67,74]. Global variation in load-bearing, gait efficiency, and terrain adaptability underscores the co-evolution of functional traits with human use and environmental context.

### 4.3. Meat and Milk Production Profiles

Donkey meat composition is remarkably conserved worldwide (Table 3), featuring high protein, low fat, and favorable fatty acid profiles [75,76,77,78,79]. Nevertheless, production systems strongly shape carcass traits [80]. European intensive systems (e.g., Italy) slaughter foals around 8 months, producing standardized carcasses with controlled fat and glycogen content, while Mediterranean and North African semi-extensive systems rely on grazing and adult slaughter, yielding leaner, more variable meat [43,81,82]. Chinese large-bodied breeds, under integrated industrial management, combine efficient growth and dual-purpose production, achieving market traits by 7 months while retaining adaptive capacities [83,84,85].

Milk production follows similar patterns (Table 4). Across regions, yields average 1–2 kg/day, with conserved low fat, moderate protein, and high lactose [90,91,92,93]. European and Balkan populations are managed for specialized dairy products, emphasizing standardization and quality, whereas Asian populations optimize yield and composition via dietary adjustments and management [94,95,96,97,98,99,100,101]. Comparative data indicate that breeds from China, India, Italy, and North Africa share similar milk nutrient profiles, suggesting strong conservation of lactation traits despite divergent management [102,103,104]. Regional differences thus reflect production strategy rather than innate biological limits.

### 4.4. Regional Differentiation in Ecological Adaptation and Disease Resistance Profiles

Donkey populations across global regions exhibit contrasting ecological adaptation strategies, largely reflecting the specific climatic pressures under which they evolved. Populations from cold or high-altitude zones commonly show enhanced hypoxia tolerance and cold resistance, supported by genetic signals such as selection at EGLN1 [109]. By comparison, North African and West Asian donkeys are shaped by extreme heat and water scarcity, displaying strong thermoregulation and dehydration tolerance [110]. European breeds, however, often lack these climatic adaptations and require substantial management support in cold–humid environments [29,111]. In Latin America and Australia, both domestic and feral donkeys demonstrate broad ecological plasticity, maintaining function under thermal extremes and heavy parasitic exposure, which partly explains their success as invasive species in certain ecosystems [112,113,114,115,116].

These ecological differences extend directly into nutritional adaptation. Breeds from resource-limited systems—such as those in Africa and the Middle East—prioritize metabolic efficiency under low-input grazing conditions [110,117]. Conversely, dairy-oriented European populations maintain stable milk yield only under intensive nutritional support [35,118]. Populations in East Asia, particularly under structured total mixed ration (TMR) systems, show rapid performance responses to improved nutrition [119,120,121], whereas feral donkeys in South America and Australia exhibit metabolic downregulation and microbiome-driven nutrient extraction strategies to survive in nutrient-poor environments [122]. Thus, regional feeding systems and long-term environmental selection jointly reinforce divergent nutritional ecotypes across global donkey populations.

**Figure 3:**
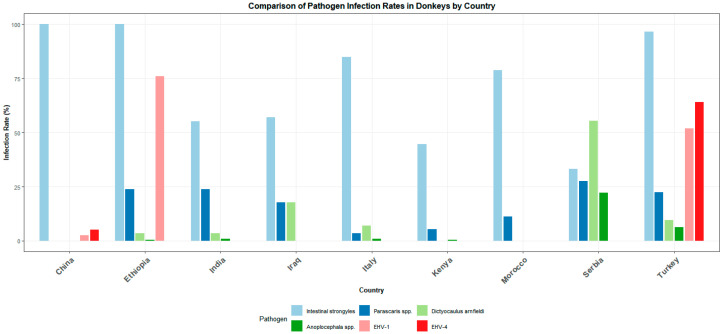
Cross-national comparative analysis of gastrointestinal parasitic infection prevalence and herpesvirus seroprevalence in global donkey populations [133,139,140,141,142,143,144,145,146,147,148,149,150].

Differences in disease resistance further mirror these ecological and nutritional divides. Regions relying on extensive, low-input management—especially Africa and Latin America—show the highest gastrointestinal parasite burdens and the most rapid emergence of anthelmintic resistance [123,124,125,126] (Figure 3). European countries report widespread resistance as well, though under more structured surveillance programs [127,128,129]. Viral and bacterial exposure patterns similarly follow regional environmental pressures: equine herpesvirus (EHV-1/4) is prevalent in European, North African, and Balkan donkeys [57,130,131,132], with recent epidemiological reports emerging from China [133,134]. Zoonotic infections such as toxoplasmosis, brucellosis, and leptospirosis reach their highest seroprevalence in Mediterranean and Latin American populations [135,136,137,138], contrasting with lower exposure in more intensively managed systems.

## 5. Comparative Analysis of Industrial Development Models and Strategic Trajectories

**Figure 4:**
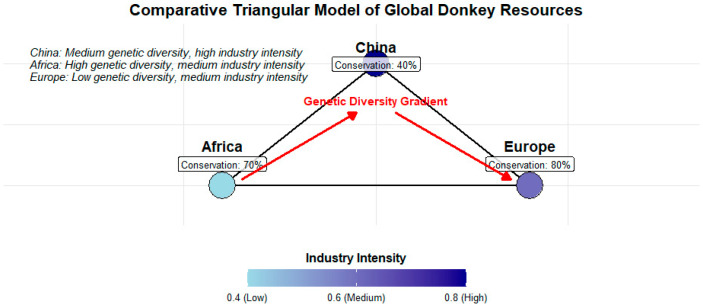
Triangular comparative framework illustrating the relationships among regional genetic diversity indices, industrial production intensity, and conservation status of global donkey genetic resources.

The global donkey industry is shifting from traditional, single-purpose systems toward diversified, sustainability-oriented production frameworks [151] (Figure 4). This transition reflects the growing need to balance market expansion, welfare expectations, and conservation responsibilities. Countries that have adopted regulated production chains, structured breeding programs, and transparent product standards are progressing more effectively toward stable industrialization.

In China, industrial upgrading focuses on integrating dairy-oriented breeding lines, standardized selection programs, and regionally differentiated conservation units, representing a model that combines genetic preservation with commercial expansion [9,10,152,153,154]. In Europe and North America, the sector operates under highly regulated, welfare-centered systems in which product traceability, protected designations, and advanced biotechnologies—such as genomic tools, semen cryopreservation, and reproductive interventions—support both market development and long-term breed conservation [155,156,157,158,159,160,161,162,163,164,165,166,167,168,169]. These contrasting models illustrate region-specific pathways toward sustainable industrial growth.

Despite regional diversity, global development converges around three strategic priorities: (1) diversification of production systems to enhance resilience and safeguard genetic heterogeneity [158]; (2) strengthening of governance and regulatory capacity to ensure welfare compliance and genetic integrity [156,157]; and (3) integration of conservation science into industrial planning through genomic selection, germplasm repositories, and structured breeding networks [155,164,165,166]. Together, these strategies provide a coherent framework for steering the global donkey industry toward long-term sustainability.

## 6. Critical Challenges Confronting the Global Donkey Industry

Despite ongoing industrial transformation, the global donkey sector faces region-specific constraints that impede sustainable development [170,171,172]. In high-income regions such as Europe and North America, limited production scale, stringent welfare regulations, and the niche nature of donkey-derived products restrict profitability and slow genetic improvement efforts [91,154,169]. Compliance costs and the need for standardized quality frameworks remain persistent barriers.

In sub-Saharan Africa and Latin America, donkeys continue to underpin smallholder livelihoods, yet inadequate veterinary infrastructure, widespread informal slaughter, and livestock theft have accelerated population declines [173,174,175,176]. These pressures are amplified by rising international demand, which can destabilize local availability and increase socioeconomic vulnerability.

Meanwhile, rapid industrial expansion in parts of Asia has intensified the tension between market demand and conservation needs [177]. Declining domestic herd sizes and reliance on complex cross-border supply chains introduce biosecurity risks and raise sustainability concerns, particularly where regulatory oversight is weak [178,179,180].

Across all regions, reproductive inefficiency—including irregular estrous cycles, low conception rates, and long gestation periods—remains a universal biological bottleneck limiting herd recovery [181,182,183]. Compounding these issues is a pronounced global research gap: compared with other livestock species, donkeys receive far less investment in welfare science, reproductive biotechnology, and genetic improvement research, leaving fundamental constraints insufficiently addressed [184].

## 7. Future Development Trajectories for China’s Donkey Industry

Agricultural modernization and mechanization have sharply reduced donkey populations in China, leading to the erosion of indigenous purebred genetic resources. As irreplaceable biological assets shaped by long-term ecological adaptation and selective breeding, these populations require coordinated characterization and conservation, supported by national genetic surveys and state-managed breeding centers [9,151,185]. While intensive production systems improve operational efficiency and resource utilization, further research is needed to optimize nutritional management, health protocols, and productivity in meat- and dairy-oriented production models [17,186]. Incorporating advanced technologies from the dairy cattle sector offers potential to enhance milk yield, product quality, and overall system performance.

Simultaneously, genomic technologies—including whole-genome sequencing, high-density SNP arrays, and genome-based selection algorithms—are enabling a shift from traditional phenotypic selection toward precise molecular breeding. These tools allow accurate assessment of genetic diversity, inbreeding, and loci linked to key economic traits, supporting early selection for growth, milk yield, and disease resistance, and accelerating the development of specialized lines such as dairy-focused populations [22]. Genomic insights also facilitate the identification of vulnerable lineages, monitoring of genetic erosion, and optimization of mating schemes, enhancing industrial efficiency while maintaining long-term genetic health [17,151,186,187].

Future industrial transformation should therefore emphasize: (i) standardized, large-scale dairy production systems with integrated quality assurance [111,187]; (ii) genomic-assisted breeding programs for specialized production lines [22]; (iii) diversification into companion, therapeutic, and eco-tourism applications to improve resilience and value creation [168]; and (iv) regulated biomedical utilization frameworks that balance expansion with welfare and conservation imperatives [105]. Together, these strategies shift the industry from resource-extractive practices toward innovation-driven, value-added, and genetically informed development.

## 8. Conclusions

Sustaining global donkey populations requires a strategic shift from trait-by-trait improvement to a systems perspective that integrates genetics, production ecology, and long-term resource management. Future progress will depend on strengthening population resilience, prioritizing adaptive capacity, and building production environments that do not erode the limited genetic base of many regional breeds. Coordinated policies, data-guided breeding goals, and investment in welfare and husbandry infrastructure are essential to secure both the biological and economic future of donkey industries.

## Figures and Tables

**Table 1:** Geographic distribution and classification of donkey breeds in China.

Breeds	Size [Weight (kg)]	Size [Height (cm)]	Usage	Location
Taihang donkey	Male: 152.66	Male: 114.70 ± 8.64	Meat and draft	Hebei Province
	Female: 139.49	Female: 104.22 ± 7.26		
Yangyuan donkey	Male: 300.37	Male: 133.60 ± 5.06	Meat and draft	Hebei Province
	Female: 228.41	Female: 123.10 ± 8.44		
Guangling donkey	Male: 335.20	Male: 141.40 ± 2.50	Excellent donkey species for breeding large mules	Shanxi Province
	Female: 331.67	Female: 139.30 ± 3.80		
Jinnan donkey	Male: 276.34	Male: 133.22 ± 3.73	Meat and draft	Shanxi Province
	Female: 276.56	Female: 133.16 ± 3.50		
Linxian donkey	Male: 262.65	Male: 124.00	draft breed	Shanxi Province
	Female: 252.63	Female: 123.60 ± 3.00		
Kulun donkey	Male: 184.59	Male: 121.20 ± 1.93	draft breed	Inner Mongolia
	Female: 150.54	Female: 110.12 ± 2.36		
Biyang donkey	Male: 285.80	Male: 138.70 ± 5.40	Meat and draft	Henan Province
	Female: 263.00	Female: 131.40 ± 5.20		
Qingyang donkey	Male: 273.55	Male: 129.41 ± 2.52	Meat and draft	Gansu Province
	Female: 242.65	Female: 124.93 ± 2.78		
Subei donkey	Male: 196.98	Male: 122.60 ± 7.10	Meat and draft	Jiangsu Province
	Female: 184.23	Female: 118.40 ± 6.00		
Huaibei Gray donkey	Male: 172.94	Male: 116.12 ± 3.45	Meat and draft	Anhui Province
	Female: 148.87	Female: 109.30 ± 4.89		
Dezhou donkey	Male: 285.93	Male: 140.22 ± 3.80	Meat, milk, and draft	Shandong Province
	Female: 261.31	Female: 135.03 ± 4.76		
Changyuan donkey	Male: 251.80	Male: 136.00 ± 3.40	Meat and draft	Henan Province
	Female: 235.10	Female: 129.40 ± 4.70		
Chuan donkey	Male: 124.78	Male: 98.73 ± 5.32	Meat and draft	Sichuan Province
	Female: 104.61	Female: 95.44 ± 4.28		
Yunnan donkey	Male: 127.27	Male: 102.30 ± 5.72	Meat and draft	Yunnan Province
	Female: 119.39	Female: 98.89 ± 4.42		
Tibetan donkey	Male: 128.39	Male: 102.86 ± 4.50	Draft and the biological hormone production function	Tibet
	Female: 128.47	Female: 106.13 ± 8.50		
Guanzhong donkey	Male: 254.77	Male: 133.45 ± 2.11	Meat and draft	Shaanxi Province
	Female: 228.37	Female: 128.12 ± 4.82		
Jiami donkey	Male: 245.30	Male: 126.80 ± 3.70	Meat and draft	Shaanxi Province
	Female: 238.90	Female: 124.10 ± 3.70		
Shanbei donkey	Male: 155.29	Male: 115.65 ± 5.40	Meat and draft	Shaanxi Province
	Female: 145.88	Female: 110.81 ± 5.69		
Liangzhou donkey	Male: 154.72	Male: 108.90 ± 6.39	Meat and draft	Gansu Province
	Female: 141.20	Female: 109.93 ± 8.63		
Qinghai donkey	Male: 123.02	Male: 101.90 ± 9.43	Meat and draft	Qinghai Province
	Female: 110.91	Female: 99.76 ± 7.51		
Xiji donkey	Male: 211.78	Male: 124.30 ± 4.60	Meat and draft	Ningxia Province
	Female: 215.67	Female: 123.30 ± 6.10		
Hetian Gray donkey	Male: 255.65	Male: 132.00 ± 1.70	Meat and draft	Xinjiang Province
	Female: 246.49	Female: 130.10 ± 3.33		
Turfan donkey	Male: 316.73	Male: 141.20 ± 5.65	Meat and draft	Xinjiang Province
	Female: 302.46	Female: 135.54 ± 4.82		
Xinjiang donkey	Male: 181.30 ± 36.00	Male: 116.00 ± 9.40	Meat and milk performance	Xinjiang Province
	Female: 156.00 ± 31.10	Female: 107.70 ± 7.30		

These breeds and their location information are obtained from the National Catalog of Livestock and Poultry Genetic Resources: https://zypc.nahs.org.cn/pzml/classify.html; (accessed 12 August 2025).

**Table 2:** The morphology and production characteristics of representative international donkey breeds.

Breeds	Size [Weight (kg)]	Size [Height (cm)]	Usage	Location
Graciosa donkey	Male: 157.84	Male: 106.53	draft animals	Portugal [32]
	Female: 172.77	Female: 106.55		
Martina Franca	Male: 331.72	Male: 144.94	Milk and hybrid improvement	Italy [33]
	Female: 331.42	Female: 137.59		
Montanaro	Male: 396.00	Male: 143.90	Dairy donkey and draft animals	Italy [33]
	Female: 404.00	Female: 142.70		
Ragusano donkey	Male: 325.00	Male: 138.00	Dairy-based	Italy [34,35]
	Female: 320.00	Female: 130.00		
Amiata donkey	Male: 200.00	Male: 140.00	Combination of dairy and eco-tourism	Italy [7]
	Female: 150.00	Female: 135.00		
Andalusian Donkey	Male: 400.00	Male: 148.00	draft animals and producing mules	Spain [7]
	Female: 370.00	Female: 145.00		
Catalonian Donkey	Male: 400.00	Male: 142.00	Traditional service and piggybacking services	Spain [7]
	Female: 350.00	Female: 136.00		
Omo Donkey	Male: 169.90	Male: 108.65	Transportation and piggybacking, Dairy donkey	Ethiopia [36]
	Female: 158.70	Female: 108.42		
Baudet du Poitou	Male/Female: 346.40	Male/Female: 139.70	Dairy donkey and cultural symbols	France [37]
Sindhi donkey	Male: 84.95	Male: 98.80	Carrying and producing donkey milk	Pakistan [38]
	Female: 89.54	Female: 97.93		
Andhra type donkey	Male: 80.14	Male: 94.57	Carrying and breeding mules	India [38]
	Female: 73.69	Female: 89.82		
Spiti donkey	Male: 75.12	Male: 88.59	Animal packing	India [38]
	Female: 75.69	Female: 88.65		
American Mammoth Jackstock	Male/Female: 430.00	Male/Female: 143.00	Service, ornamental, and mule breeding	USA [39]
Miniature Mediterranean donkey	Male/Female: 180.00	Male/Female: 92.00	Pet donkey	USA [39]
Banat donkey	Male: 219.00	Male: 126.00	Functional service animals	Serbia [40]
	Female: 208.7	Female: 117.90		
Balkan donkey	Male: 250.00	Male: 104.90	Dairy donkey/draft donkey	Serbia [26,41]
	Female: 200.00	Female: 95.00		
Brick kiln donkeys (non-varieties)	Male/Female: 186.00	Male/Female: 115.00	Milk and service use	Egypt [42]
Moroccan donkey	Male/Female: 252.00	Male/Female: 129.00	Milk and service use	Morocco [43]

**Table 3:** Comparison of nutritional composition between donkey meat and beef.

Nutrient Composition Index	Chinese Donkey Meat [86]	Martina Franca Meat [87]	Beef [88,89]
Protein (g/100 g)	21.5	22.3	21.7
Fat (g/100 g)	3.2	2.4	11.6
Moisture (%)	73.8	74.8	65.9
Cholesterol (mg/100 g)	74	67.4	55.4
Ash (%)	1.1	1.0	0.9
Calcium (mg/100 g)	2.0	8.6	2.8
Phosphorus (mg/100 g)	178.0	212.0	186.0
Potassium (mg/100 g)	325.0	343.0	289.0
Sodium (mg/100 g)	46.9	52.5	63.4
Magnesium (mg/100 g)	7.0	24.0	21.8
Iron (mg/100 g)	4.3	3.8	1.3
Zinc (mg/100 g)	4.3	3.7	2.3
Total saturated fatty acid (%)	41.7	41.1	54.1
Total monounsaturated fatty acid (%)	38.7	33.8	42.8
Total polyunsaturated fatty acid (%)	19.5	25.2	3.0

**Table 4:** Comparison of donkey milk components.

Donkey Milk Index	Ragusana [35]	China Donkey [99]	Indian Donkey [102,105,106]	Serbian Donkey [107,108]
Milk yield (kg/day)	1.64	1.14	1.00	1.00
Protein (g/100 g)	1.34	1.65	2.03	1.69
Fat (g/100 g)	0.16	0.20	0.86	0.41
Lactose (g/100 g)	6.07	6.55	5.75	5.83
Ash (g/100 g)	0.36	0.38	0.51	0.37
Dry matter (g/100 g)	8.19	9.55	9.61	8.49
Calcium (mg/100 g)	54.36	87.23	61.25	65.56
Phosphorus (mg/100 g)	43.44	62.44	32.99	53.33
Magnesium (mg/100 g)	6.13	7.38	7.27	8.44
Sodium (mg/100 g)	43.77	13.79	13.84	44.44
Potassium (mg/100 g)	110.27	67.97	47.39	71.11
Zinc (mg/kg)	2.24	0.01	1.83	2.19

## Data Availability

Breed and phenotype information were obtained from the (https://zypc.nahs.org.cn/pzml/classify.html, accessed 12 August 2025) and the FAO Domestic Animal Diversity Information System (DAD-IS) (https://www.fao.org/dad-is/browse-by-country-and-species/en/, accessed 12 August 2025).
